# Regulation of degenerative spheroids after injury

**DOI:** 10.1038/s41598-020-71906-x

**Published:** 2020-09-22

**Authors:** Yu Yong, Kanchana Gamage, Courtny Cushman, Anthony Spano, Christopher Deppmann

**Affiliations:** 1 Department of Biology, University of Virginia, Charlottesville, VA 22904-4328 USA; 2 Amgen, Massachusetts and Department of Stem Cell and Regenerative Biology, Harvard University, Cambridge, MA 02138 USA; 3 Department of Neuroscience and Biomedical Engineering, University of Virginia, Charlottesville, VA 22904-4328 USA; 4 Department of Cell Biology, University of Virginia, Charlottesville, VA 22904-4328 USA

**Keywords:** Cellular neuroscience, Molecular neuroscience, Peripheral nervous system, Neurodegeneration

## Abstract

Neuronal injury leads to rapid, programmed disintegration of axons distal to the site of lesion. Much like other forms of axon degeneration (e.g. developmental pruning, toxic insult from neurodegenerative disorder), Wallerian degeneration associated with injury is preceded by spheroid formation along axons. The mechanisms by which injury leads to formation of spheroids and whether these spheroids have a functional role in degeneration remain elusive. Here, using neonatal mouse primary sympathetic neurons, we investigate the roles of players previously implicated in the progression of Wallerian degeneration in injury-induced spheroid formation. We find that intra-axonal calcium flux is accompanied by actin-Rho dependent growth of calcium rich axonal spheroids that eventually rupture, releasing material to the extracellular space prior to catastrophic axon degeneration. Importantly, after injury, *Sarm1*^*−/−*^ and *DR6*^*−/−*^, but not *Wld*^*s*^ (excess NAD^+^) neurons, are capable of forming spheroids that eventually rupture, releasing their contents to the extracellular space to promote degeneration. Supplementation of exogenous NAD^+^ or expressing WLD^s^ suppresses Rho-dependent spheroid formation and degeneration in response to injury. Moreover, injured or trophically deprived *Sarm1*^*−/−*^ and *DR6*^*−/−*^, but not *Wld*^*s*^ neurons, are resistant to degeneration induced by conditioned media collected from wild-type axons after spheroid rupture. Taken together, these findings place Rho-actin and NAD^+^ upstream of spheroid formation and may suggest that other mediators of degeneration, such as DR6 and SARM1, mediate post-spheroid rupture events that lead to catastrophic axon disassembly.

## Introduction

Axons are the primary information conduits of the nervous system. Failure to maintain the integrity of axons is a feature of many neurological disorders. In response to injury, a process of axonal fragmentation, or Wallerian degeneration (WD) occurs, often resulting in permanent loss of neural function^1^. Immediately after injury, the severed axon goes through a latent phase where its overall morphology remains unchanged for 1–2 h in vitro and up to 48 h in vivo^2,3^. Intracellular calcium increases transiently during the latent phase, followed by a second global calcium wave just prior to axon fragmentation^4^. Elevation of intracellular calcium through L-type calcium channels and sodium-calcium exchanger (NCX), together with impaired mitochondrial motility and calcium buffering capacity, results in activation of the calpain protease and irreversible disassembly of the axon^5–10^. In addition to calcium flux, severed axons display cessation of axonal transport, formation of axonal swellings called spheroids, fragmentation of neurofilaments and removal of debris by recruited phagocytes. This rapid and near synchronous axonal disintegration period is called the catastrophic/execution phase of degeneration and can also be observed in developmental and other pathological regressive contexts^11–13^.

The field’s first insight into the non-passive nature of WD signaling came from the *Wld*^*s*^ mouse which harbors a neomorphic gain of function mutation and displays axon degeneration that is 10 times slower after injury compared to wild-type neurons^14,15^. The *Wld*^*s*^ gene encodes a chimeric fusion protein, consisting of the full-length nicotinamide mononucleotide adenyltransferase 1 (NMNAT1), which synthesizes NAD^+^ from its substrate nicotinamide mononucleotide (NMN), and a fragment of the ubiquitination factor UBE4B^16^. The perdurance of high NAD^+^ levels in axons is sufficient to delay Wallerian degeneration^17^. As such, depletion of NAD^+^ is known to be an important trigger for WD and is achieved in several ways after injury: (1) Turnover of NMNAT2 regulated by the ubiquitin proteasome system (UPS), palmitoylation of cysteines in NMNAT2 for membrane targeting and mitogen-activated protein kinase (MAPK) signaling^18–21^ and (2) Activation of sterile alpha and armadillo motif (SARM1), which has intrinsic NAD^+^ cleavage activity^22–24^. Beyond proteins that influence NAD^+^ levels, several other factors have been implicated in promoting Wallerian degeneration including death receptor 6 (DR6), calpain, Phr1 E3 ubiquitin ligase, dual leucine zipper kinase (DLK), c-jun n-terminal kinase (JNK), and axundead (Axed)^6,25–31^. Whether these factors all converge on similar signaling hubs such as NAD^+^ remains to be determined.

Because the cytoskeleton is crucial for maintaining axon integrity, signaling pathways that promote disassembly of microtubules and actin filaments likely contribute to axon degeneration. The Rho/Rac/Cdc42 family of small G-proteins are well known for their effects on reorganization of actin, which affect cell survival, migration and vesicle trafficking^32^. In neurons, RhoA and its downstream effector Rho-associated protein kinase (ROCK) has been shown to regulate axon retraction, degeneration, regeneration and neural death in both developmental and pathological conditions^33–35^. Importantly, RhoA/ROCK has been shown to be activated in injured axons and its pharmacological inhibition delays degeneration^36,37^. Moreover, we recently found that Rho activation is required in axonal spheroid formation and degeneration triggered by trophic deprivation^10^. Whether Rho also regulates WD through mechanisms similar to developmental degeneration requires further investigation.

Formation and growth of axonal spheroids has been identified in various models of degeneration, including optic nerve injury, Alzheimer's disease, Parkinson’s disease and amyotrophic lateral sclerosis^13,38,39^. Recently, we found that these spheroids are not merely a morphological hallmark of degeneration induced by trophic withdrawal but are also functionally consequential, mediating the transition from latent to catastrophic phase^10^. Additionally, axonal spheroids arised after injury has been shown to be blocked by *Wld*^*s*^^40^. Despite a handful of descriptive reports about the content of these spheroids^13^, whether spheroid formation is influenced by the aforementioned regulators of WD (e.g. NMNATs, SARM1, DR6) remains unresolved.

Here, we demonstrate that prior to catastrophic degeneration, intra-axonal calcium increases and decreases corresponding to growth and rupture of axonal spheroids. We further demonstrate that Rho activation and changes to the actin cytoskeleton are required for spheroid formation after injury. We also find that consistent with previous observations, neurons derived from *Wld*^*s*^ mice display impaired spheroid formation and rupture after injury^40^. We show that upstream of Rho activation, both exogenous supplementation of NAD^+^ and the presence of a more stable axonally targeted NMNAT, such as WLD^s^, suppress spheroid formation. In contrast, loss of *DR6* or *SARM1* has minimal effect on spheroid formation and rupture after injury. Moreover, we find that SARM1 and DR6, but not WLD^s^ are required for responding to spheroid-derived pro-degenerative cues to promote catastrophic degeneration. These findings indicate a separable role of classic Wallerian degeneration effectors with respect to spheroid formation after axotomy.

## Results

### Intra-axonal calcium increases in enucleated axons and accumulates in spheroids prior to catastrophic degeneration

**Figure 1:**
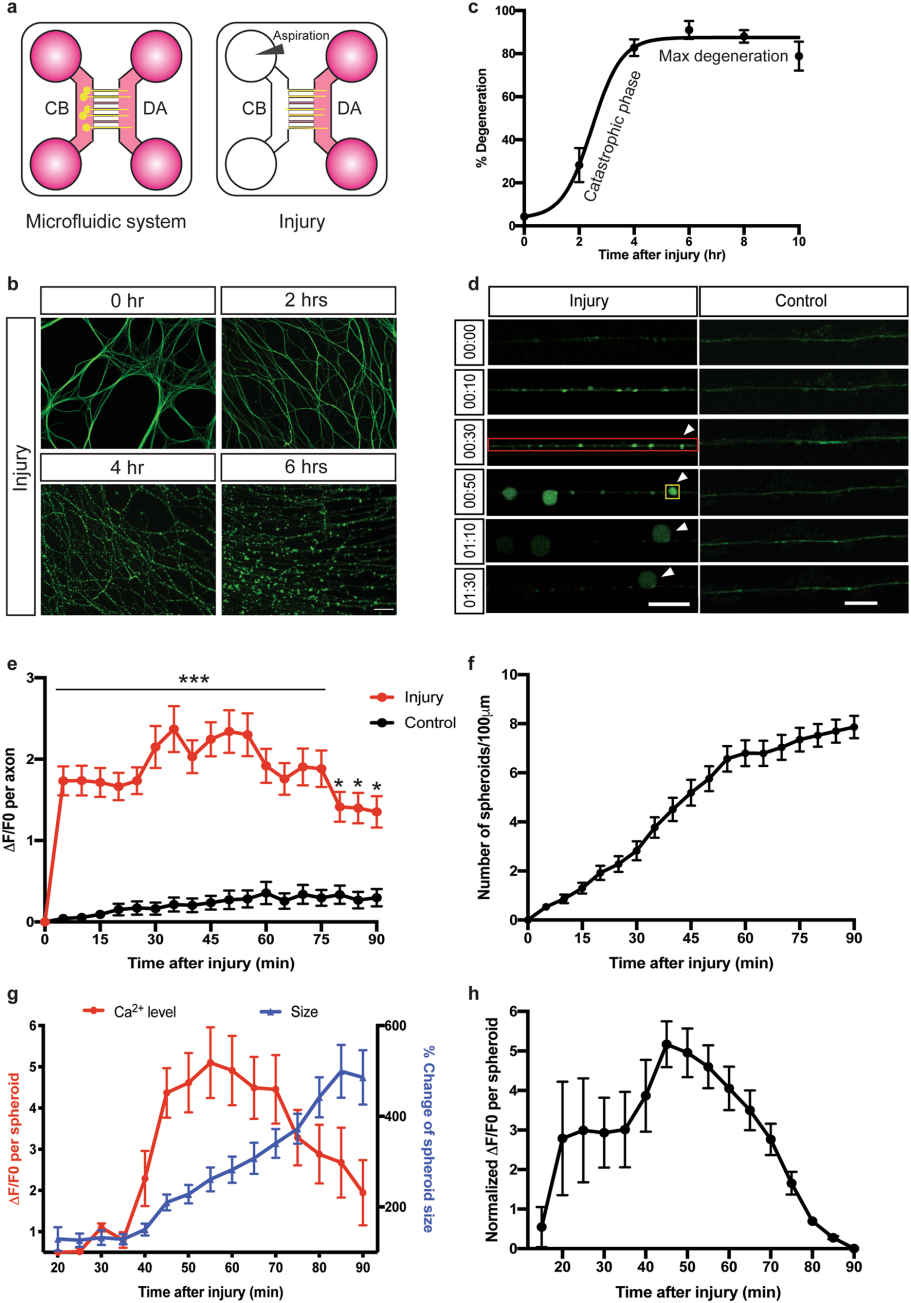
Axoplasmic calcium dynamics and formation of spheroids prior to catastrophic degeneration in response to injury. (**a**) Schematic representation of injury paradigm in microfluidic devices. Cell bodies (CB) and distal axons (DA) are seperated. All the cultures were maintained in the presence of 45 ng/mL NGF. For the “injury” condition, neurons were enucleated by aspiration in PBS. (**b**) Representative images of β3-tubulin immuno-stained distal sympathetic axons before treatment (0 h), 2, 4 and 6 h after injury. Scale bar = 50 µm. (**c**) Degeneration time course after injury. Catastrophic phase and maximum of degeneration are noted. Nonlinear regression curve was drawn according to the Hill equation. *n* = 3 for each time point. (**d**) Fluo4-AM calcium imaging of sympathetic axons at the indicated times after injury. For the “injury” condition, neurons were enucleated by aspiration in PBS and then incubated with Fluo4-AM for calcium imaging. For the “Control” condition, no injury was performed. Red box indicates the individual axon as a region of interest. Yellow box indicates axonal spheroid as a region of interest. White arrowheads indicate the formation and growth of spheroid. Scale bar = 10 µm. (**e**) Calcium fluorescence change of control or injured axons over time. Total number of *n* = 76 (injury) and *n* = 30 (control) of axons from 3 independent litters were quantified. (**f**) Quantification of axonal spheroid number per 100 µm of axon at the indicated times after injury. Total number of *n* = 47 axons from 3 independent litters were counted. (**g**) Calcium fluorescence and size change of axonal spheroid at the indicated times after injury. Total number of *n* = 14 axonal spheroids from 3 independent litters were quantified. (**h**) Quantification of normalized calcium fluorescence of axonal spheroids from 20 to 90 min after injury. Individual axonal spheroids were quantified: *n* = 14 spheroids from 3 independent replicates. Data are reported as mean ± SEM, *p < 0.05; ***p < 0.0001, two-way ANOVA with Sidak’s multiparisons test.

We first sought to determine whether calcium accumulates in spheroids after injury as we have observed previously for degeneration associated with trophic deprivation. To this end, we cultured mouse sympathetic neurons in microfluidic devices, which separate soma and axons (Fig. 1a). Cell bodies of sympathetic neurons were enucleated by aspiration of the cell body chamber in PBS, which leaves axons residing in the distal axon chamber and microgrooves intact. After injury, these axons remain intact for roughly an hour as measured by microtubule integrity (β3-tubulin staining). At the conclusion of the latent phase, the majority of axons rapidly degenerate, going from 7.5 ± 2.08% to 82.75 ± 3.9% of degeneration within 90 min (Fig. 1b,c). This is referred to as the catastrophic or execution phase of degeneration^41^. We applied the Fluo4-AM calcium dye to axons 30 min prior to imaging and recorded the calcium dynamics for 90 min after injury (Fig. 1d). For injured axons, intra-axonal calcium had a roughly twofold increase from baseline prior to onset of the catastrophic phase (Fig. 1e). Much of this calcium was concentrated in nascent spheroids (Fig. 1d), consistent with previous in vivo and in vitro injury studies^40,42^ and similar to our recent findings in the context of trophic withdrawal^10^. After observing the initial formation of calcium rich spheroids, these structures increase in size from 3.2 ± 0.4 to 13 ± 1.2 μm^2^ (roughly 400%) between 20 and 80 min after injury. Spheroidal calcium levels increased by roughly fivefold at 1 h after injury (Fig. 1g). Interestingly, spheroidal calcium levels decrease as spheroids increase in size. Besides the increase in size and calcium level in individual spheroids, the number of spheroids increased from 0.54 ± 0.14 to 6.8 ± 0.53 per 100 μm of axon between 5 and 60 min after injury (Fig. 1f). We also examined spheroidal calcium as a function of spheroidal area and found the same trend (Fig. 1h). Taken together, these results indicate that the formation of calcium rich axonal spheroids is a morphological hallmark that occurs prior to entry into the catastrophic phase of degeneration after injury.

### Formation of axonal spheroids requires Rho activation and actin remodeling

**Figure 2:**
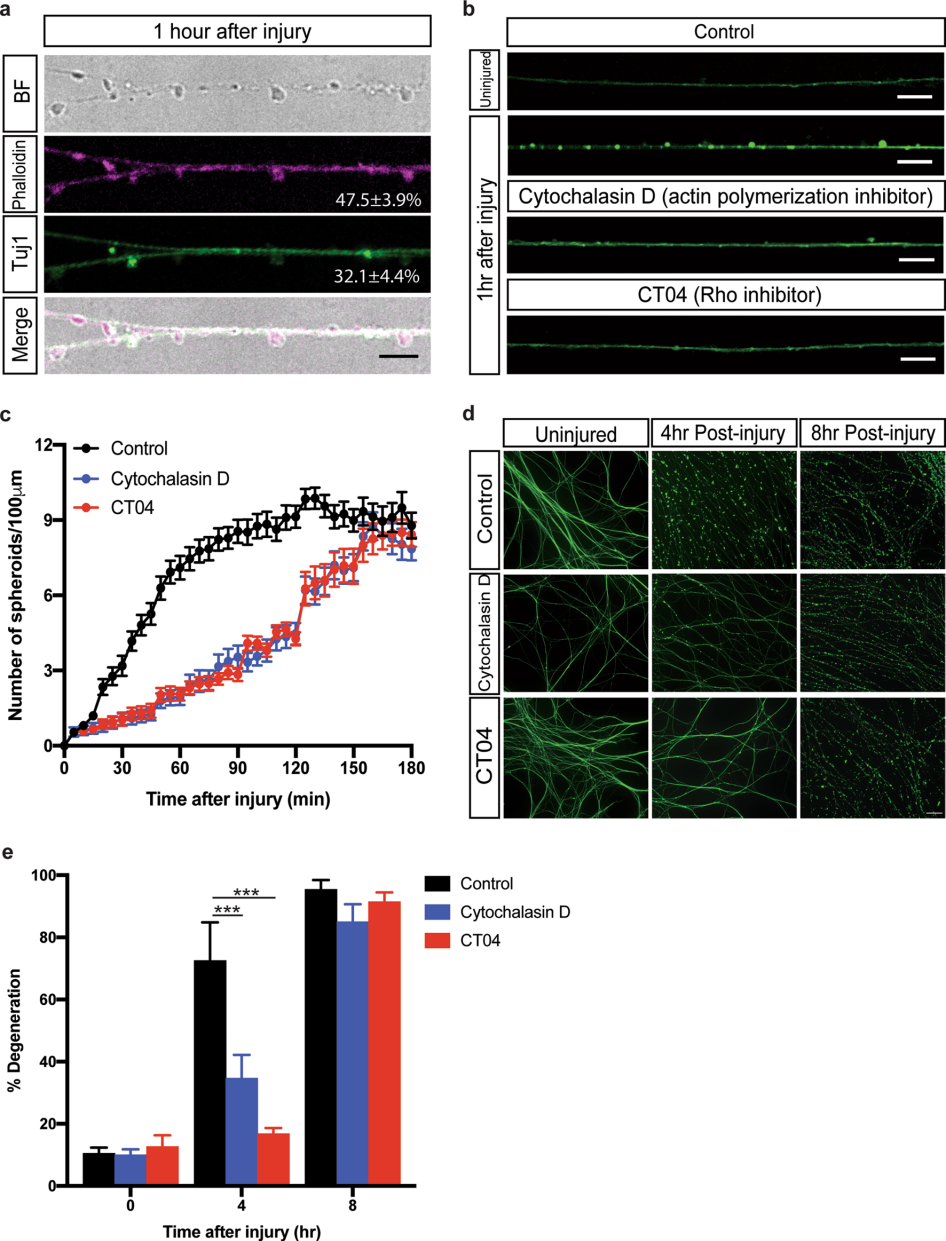
Rho activation and actin remodeling are required for axonal spheroid formation in response to injury. (**a**) Representative axons/spheroids visualized for bright field, Phalloidin and β3-tubulin (Tuj1) 1 h after injury. Scale bar = 5 μm. Percentages of Phalloidin positive and Tuj1 positive spheroids were quantified next to the images, respectively. Total number of *n* = 10 axons were quantified. (**b**) Fluo4-AM calcium imaging of wild-type sympathetic axons with or without drug treatment. For the “CT04” group, wild-type axons were incubated in SCG media containing 1 µg/mL Rho inhibitor CT04, for 2 h prior to injury. For the “Cytochalasin D” group, wild-type axons were incubated in SCG media containing 10 µg/mL actin polymerization inhibitor for 2 h prior to injury. Scale bar = 10 µm. (**c**) Quantification of axonal spheroid number per 100 µm of wild-type sympathetic axons at the indicated times after injury in the absence and presence of CT04 or Cytochalasin D. Total number of *n* = 50 (control), *n* = 28 (cytochalasin D), *n* = 32 (CT04) axons from cultured neurons harvested from 3 independent litters were quantified. (**d**) Representative images and (**e**) quantification of degeneration of wild-type distal sympathetic axons immuno-stained for β3-tubulin in the absence and presence of CT04 or Cytochalasin D. Scale bar = 50 µm. Compared to Control, 4 h post-injury (*n* = 4), *p* < 0.0001, *n* = 6 for Cytochalasin D, 4 h post-injury; *p* < 0.0001, *n* = 5 for CT04, 4 h post-injury, two-way ANOVA with Dunnett’s multiple comparisons test. Data are reported as mean ± SEM, *p < 0.05; ***p < 0.0001.

What signaling pathways trigger axonal spheroid formation after injury? Given the dramatic outgrowth of membrane, we speculated that spheroid formation may involve cytoskeletal remodeling, similar to what we observed after trophic deprivation^10^. To examine actin and β3-tubulin abundance in spheroids we stained enucleated axons with phalloidin and Tuj1 1 h after injury. We found that actin accumulated in 47.5 ± 3.9% of the spheroids examined, while β3-tubulin accumulated in 32.1 ± 4.4% of spheroids (Fig. 2a). We next sought to determine whether the formation of spheroids involves actin remodeling. To examine this, cultures were pre-treated (3hrs prior to injury) with the actin polymerization inhibitor cytochalasin D (10 μg/μL), which delayed spheroid formation (Fig. 2b,c). Rho activation is known to influence actin assembly^43^, and may serve as a molecular switch to govern spheroid formation. Indeed, inhibiting Rho family members using the C3 transferase, CT04 (1 μg/mL, 2 h prior to injury), also suppressed spheroid formation (Fig. 2b,c). Interestingly, pretreatment with cytochalasin D or CT04 delayed degeneration for up to 4 h after injury (Fig. 2d,e).

### Axonal spheroids develop membrane rupture after injury

**Figure 3:**
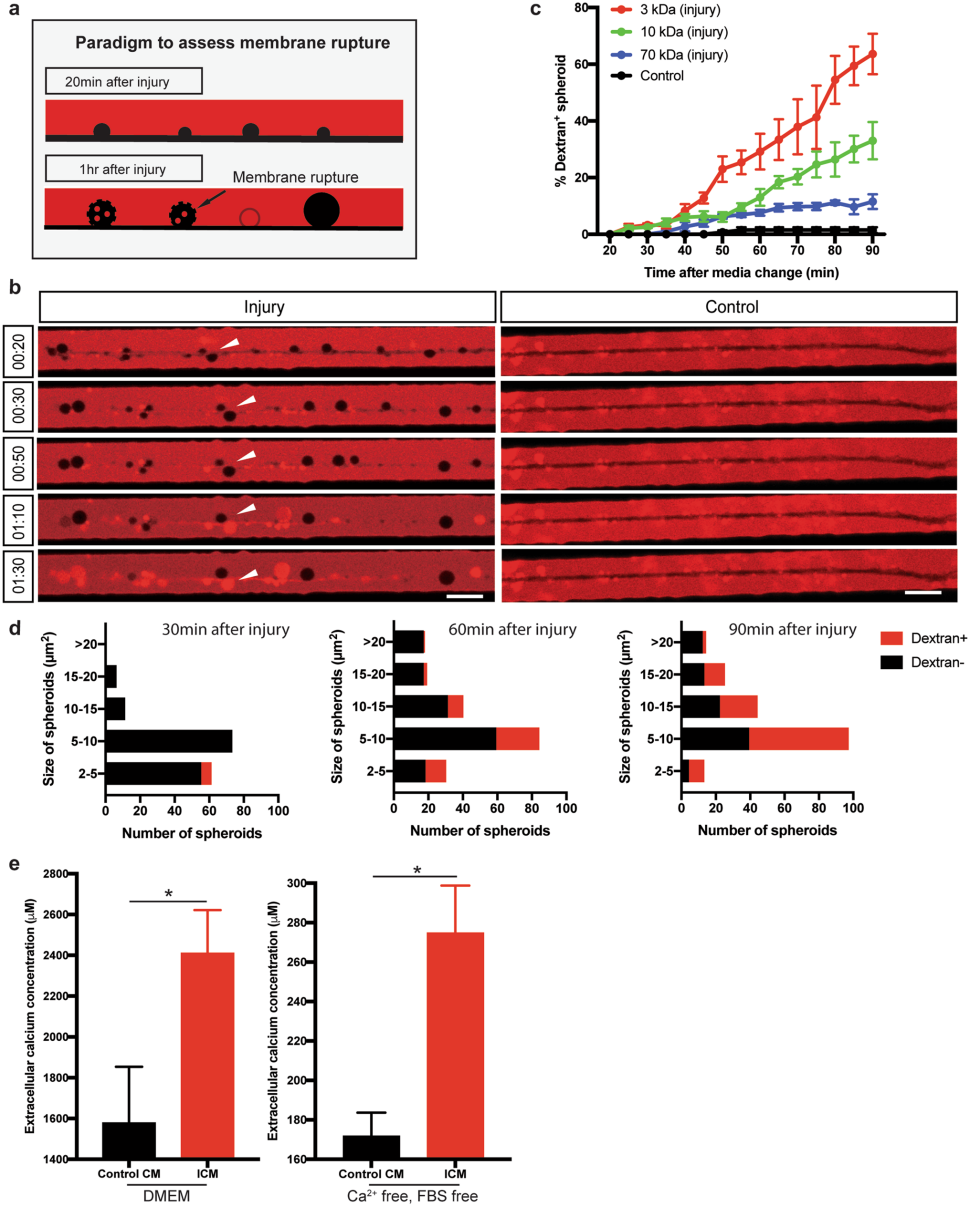
Axonal spheroids develop membrane rupture after injury. (**a**) Schematic representation of the experimental paradigm to assess membrane rupture model using fluorescent dextran. 20 min after injury, fluorescent dextran (red) is not taken up by the axon (black, negative space). However, by 1 h after injury, as the plasma membrane loses integrity and ruptures, fluorescent dextran (red) can diffuse into spheroids, turning them red. Spheroids with intact membrane remain black. (**b**) Representative images of dextran 3 kDa (red) entry to axonal spheroids (black) from 20 to 90 min after injury (left column), and dextran exclusion in untreated axons (right column). White arrowheads indicate that dextran 3 kDa enter axonal spheroids 1 h after injury. Scale bar = 10 μm. (**c**) Quantification of the percentages of fluorescent 3 kDa (red), 10 kDa (green), and 70 kDa (blue) dextran positive spheroids 20–90 min after injury. Black line (control) indicates the percentages of fluorescent 3 kDa dextran positive spheroids without injury. Total number of *n* = 12 (3 kDa), *n* = 12 (10 kDa), *n* = 9 (70 kDa), and *n* = 27 (control) axons from 3 independent litters were counted. (**d**) Histogram of 3 kDa dextran negative (black) and positive (red) spheroids 30, 60, and 90 min after injury. (**e**) Measurements of extracellular calcium expelled from axons into regular SCG culture media (DMEM, left) and calcium free, FBS free media (right). In the “Control CM” group, media was collected from uninjured axons. In the “ICM” group, media was collected 1 h after injury. For DMEM groups, compared to Control CM (*n* = 4), *p* = 0.0414, *n* = 4 for ICM; For calcium free, FBS free groups, compared to Control CM (*n* = 3), *p* = 0.0185, *n* = 3 for ICM, unpaired t test. Data are reported as mean ± SEM, *p < 0.05; ***p < 0.0001.

We and others have shown that the electrical chemical gradient can be disrupted through membrane rupture on axonal spheroids in models of developmental degeneration and multiple sclerosis^10,44^. To determine whether the axonal spheroids that we observed on injured sympathetic axons develop ruptures, we bathed axons in neutral fluorescent dextran beginning 20 min after injury, which is sufficient time for the initial site of lesion to re-seal^45^. If there were any ruptures on the membrane after injury, the 3 kDa red dextran would immediately diffuse to the axoplasm (Fig. 3a). As expected, the exclusion of dextran was maintained for 40–50 min after injury, however after 50 min, the axoplasm begins to fill with fluorescent dextran (Fig. 3b). An additional movie file shows the dextran filling axonal spheroids in more detail (see Supplementary video 1). We also examined the size of ruptures using different sized dextrans. We observed that 70, 10, and 3 kDa dextran filled 11.5 ± 2.6%, 33 ± 6.6% and 63.6 ± 7.1% of axonal spheroids by 90 min after injury, respectively (Fig. 3c). This indicates permeability of small to medium sized molecules and suggests the same physiology of spheroidal rupture in WD as other degeneration paradigms. To determine whether dextran diffusion correlates with the size of spheroids, we counted the numbers and sizes of 3 kDa dextran positive and negative spheroids 30, 60, and 90 min after injury, respectively (Fig. 3d). Most of the axonal spheroids are 5–10 μm^2^ in size. However, regardless of their sizes, more spheroids develop membrane rupture at later times after injury suggesting that the probability of rupture is more impacted by time than size of spheroid.

Based on the diminution of spheroidal calcium signal 1 h after injury and the permeability of the spheroidal membrane, we speculate that intra-axonal calcium may diffuse to the extracellular space after spheroidal rupture. To test this hypothesis, we bathed axons in microfluidic devices in 100 μL of regular culture media or calcium free, serum free media and measured extracellular calcium before and 1 h after injury using a Fluo4 spectrophotometric assay^10^. For the cultures maintained in regular SCG media, calcium levels in conditioned media taken from injured axons (ICM) (2,409.51 ± 212.17 μM) was significantly higher than calcium concentration in Control CM (1578.16 ± 275.57 μM) (Fig. 3e). Additionally, we were able to observe an increase in extracellular calcium after spheroidal rupture when experiments were performed using calcium free, FBS free media (Fig. 3e). Whether or not this calcium extrusion is physiologically relevant remains to be determined. However, in this in vitro system it represents a complementary paradigm to the aforementioned dextran assay for assessing membrane integrity.

### Spheroid formation in WD deficient mutants

**Figure 4:**
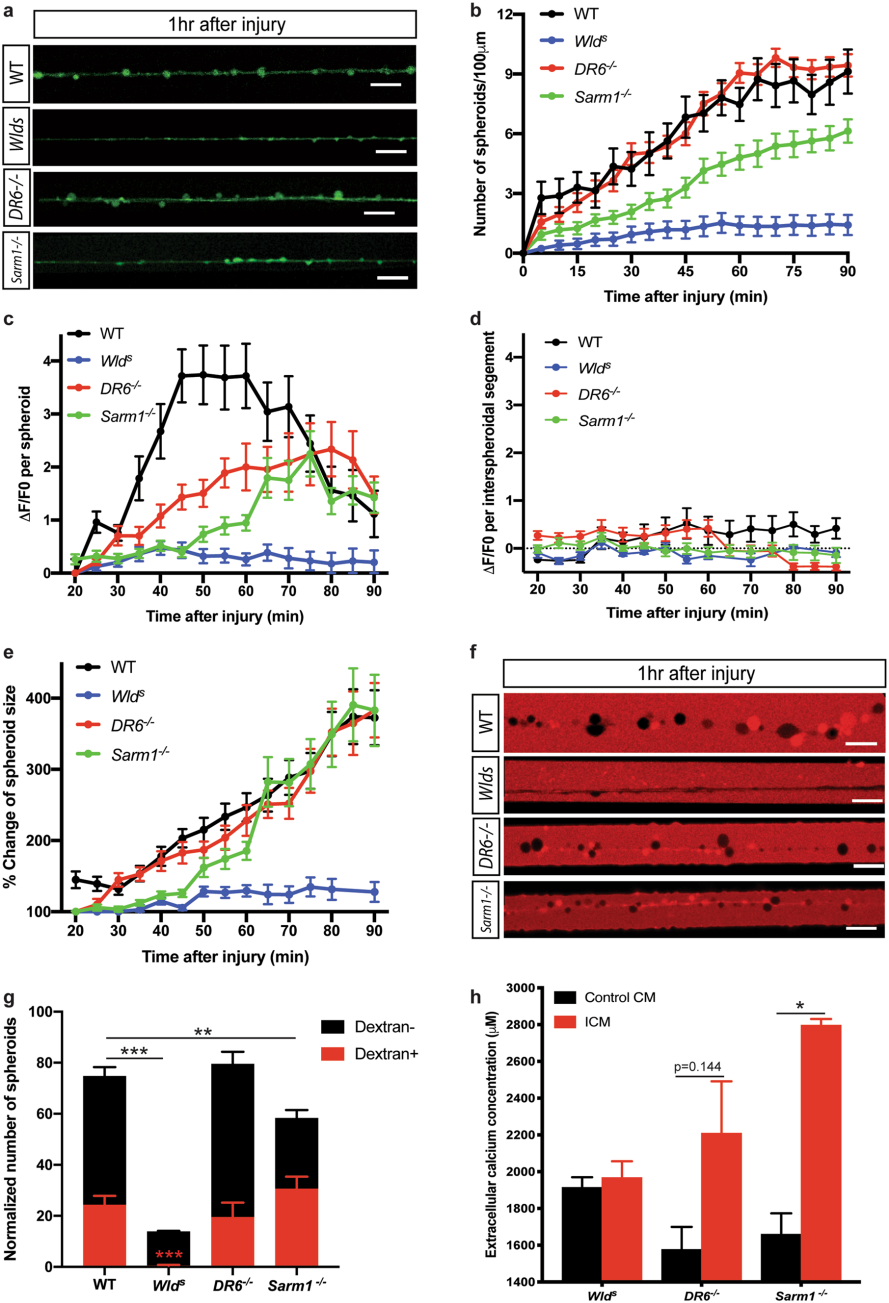
*DR6*^*−/−*^ and *Sarm1*^*−/−*^ develop axonal spheroids and spheroidal rupture after injury, while *Wld*^*s*^ does not. (**a**) Fluo4-AM calcium imaging of wild-type, *Wld*^*s*^, *DR6*^*−/−*^, and *Sarm1*^*−/−*^ sympathetic axons 1 h after injury. Scale bar = 10 µm. (**b**) Quantification of numbers of axonal spheroids, (**c**) spheroidal calcium level, (**d**) interspheroidal calcium level, and (**e**) size change of axonal spheroids on the wild-type, *Wld*^*s*^, *DR6*^*−/−*^, and *Sarm1*^*−/−*^ axons 1 h after injury, respectively. Total number of *n* = 29 (WT), *n* = 17 (*Wld*^*s*^), *n* = 57 for (*DR6*^*−/−*^), and *n* = 54 (*Sarm1*^*−/−*^) axons from 3 independent litters were counted. (**f**) Representative images of dextran 3 kDa (red) entry to axonal spheroids (black) on wild-type, *Wld*^*s*^, *DR6*^*−/−*^, and *Sarm1*^*−/−*^ axons 1 h after injury. Scale bar = 10 µm. (**g**) Normalized numbers of 3 kDa dextran negative (black) and positive (red) axonal spheroids on wild-type, *Wld*^*s*^, *DR6*^*−/−*^, and *Sarm1*^*−/−*^ axons 1 h after injury. Compared to WT, Dextran^+^, *p* < 0.0001, *n* = 21 for *Wld*^*s*^, Dextran^+^. Compared to WT, Dextran^-^, *p* < 0.0001, *n* = 22 for *Wld*^*s*^, Dextran^-^; *p* = 0.0003, *n* = 17 for *Sarm1*^*−/−*^, Dextran^-^, two-way ANOVA with Dunnett’s multiple comparisons test. (**h**) Measurement of extracellular calcium concentration in media surrounding injured and uninjured *Wld*^*s*^, *DR6*^*−/−*^ and *Sarm1*^*−/−*^ axons. All injured conditioned media was collected from distal axon chamber 1 h after injury. Compared to Control CM, *p* = 0.9982, *n* = 3 for *Wld*^*s*^, ICM; *p* = 0.1440, *n* = 6 for *DR6*^*−/−*^, ICM; *p* = 0.0141, *n* = 3 for *Sarm1*^*−/−*^, ICM, two-way ANOVA with Sidak’s multiple comparisons test. Data are reported as mean ± SEM, *p < 0.05; **p < 0.001; ***p < 0.0001.

We next examined the spheroid formation in genotypes reported to have impaired Wallerian degeneration: *Wld*^*s*^, *DR6*^*−/−*^ and *Sarm1*^*−/−*^. Calcium imaging revealed that *Wld*^*s*^ did not display a late stage calcium wave, and had a greatly diminished capacity to form spheroids in response to injury (Fig. 4a–c). This is consistent with previous observations demonstrating that mitochondria in *Wld*^*s*^ neurons have increased calcium buffering capacity and a delay in spheroid formation^4,5,7,40^. Interestingly, neurons from *DR6*^*−/−*^ and *Sarm1*^*−/−*^ mice also had attenuated spheroidal calcium compared to wild-type but significantly higher than *Wld*^*s*^ neurons (Fig. 4c). We also examined the Fluo4-AM signals in the inter-spheroidal regions of wild-type and WD mutant axons after injury. No obvious calcium wave was detected in the axons outside of spheroids after injury, suggesting that spheroids are the predominant contributor to axoplasmic calcium change (Fig. 4d). We next examined the role of WLD^s^, DR6 and SARM1 in the change in spheroid size and the accumulation of spheroids as a function of time after injury. 1 h after injury, we observed that loss of *DR6* or *SARM1* displayed a roughly 100% increase in spheroid size (Fig. 4e). Moreover, wild-type, *DR6*^*−/−*^, and *Sarm1*^*−/−*^ neurons displayed 7.5 ± 0.8, 9.1 ± 0.5, and 9.7 ± 0.7 spheroids per 100 μm of axons, respectively, whereas *Wld*^*s*^ neurons only displayed 1.4 ± 0.5 spheroids per 100 μm of axons 1 h after injury (Fig. 4b). We next examined spheroid rupture and calcium extrusion in sympathetic axons from *Wld*^*s*^, *DR6*^*−/−*^ and *Sarm1*^*−/−*^ mice. WT, *DR6*^*−/−*^ and *Sarm1*^*−/−*^ all displayed similar levels of 3 kDa dextran spheroid filling after injury, however *Wld*^*s*^ axons displayed negligible filling indicating a lack of spheroid rupture (Fig. 4f,g). Consistent with this, *Wld*^*s*^ axons showed no difference in extracellular calcium levels before or 1 h after injury, while *DR6*^*−/−*^ and *Sarm1*^*−/−*^ displayed elevation in extracellular calcium levels (Fig. 4h). Taken together, these findings suggest that WLD^s^/NMNAT is upstream of spheroid formation while the other players tested have minimal roles in this process.

### NAD^+^ acts upstream of Rho activation to suppress spheroid formation

**Figure 5:**
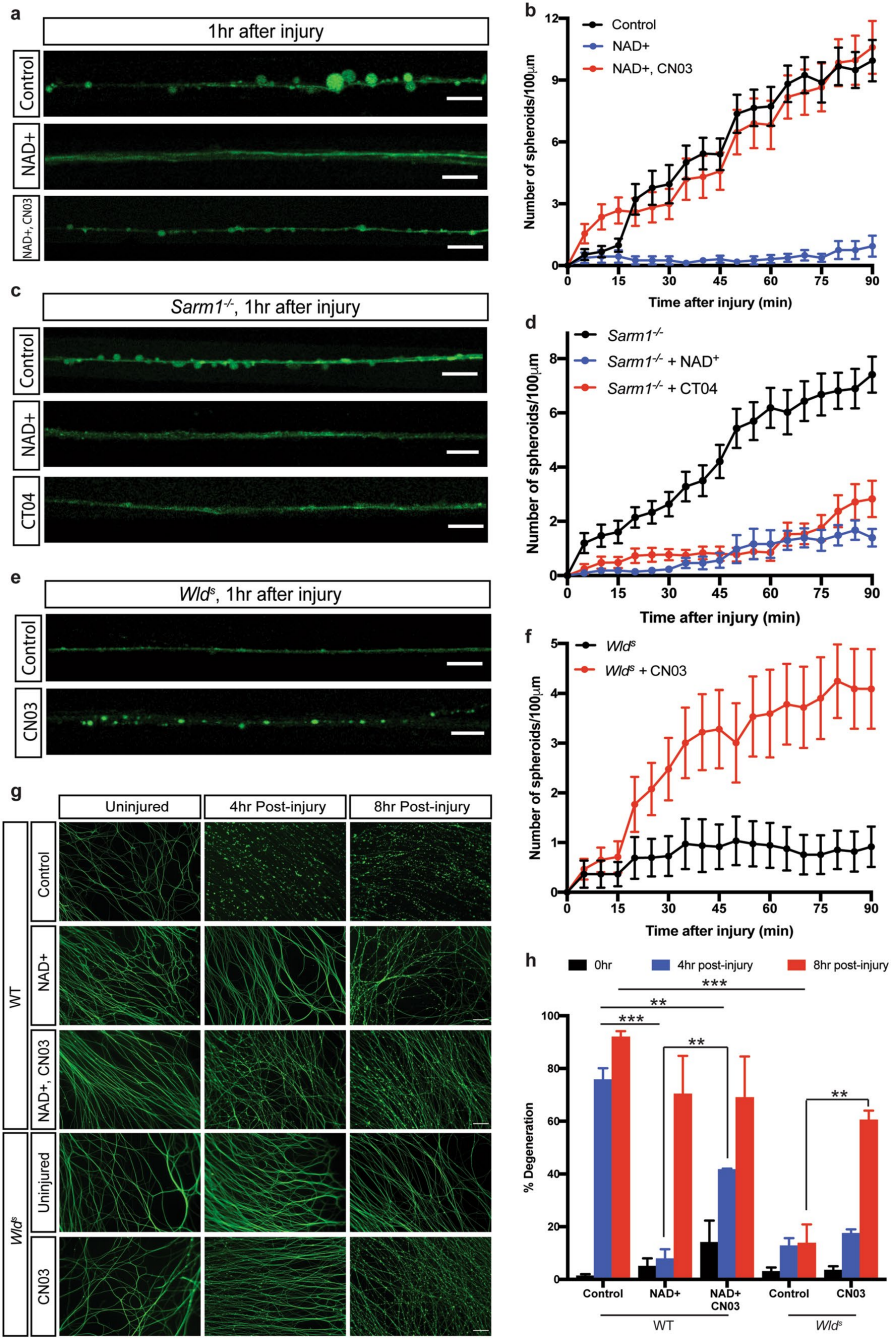
NAD^+^ acts on upstream of Rho activation to suppresses spheroid formation after injury. (**a**) Fluo4-AM calcium imaging of wild-type sympathetic axons 1 h after injury with or without drug treatment. Scale bar = 10 µm. (**b**) Quantification of axonal spheroid number per 100 µm of wild-type sympathetic axons at the indicated times after injury in the absence and presence of NAD^+^ or CN03. Total number of *n* = 23 (Control), *n* = 13 (NAD^+^), *n* = 21 (NAD^+^, CN03) axons from cultured neurons harvested from 3 independent litters were quantified. (**c**) Fluo4-AM calcium imaging of *Sarm1*^*−/−*^ sympathetic axons 1 h after injury with or without drug treatment. Scale bar = 10 µm. (**d**) Quantification of axonal spheroid number per 100 µm of *Sarm1*^*−/−*^ sympathetic axons at the indicated times after injury in the absence and presence of NAD^+^ or CT04. Total number of *n* = 30 (Control), *n* = 16 (NAD^+^), *n* = 25 (CT04) *Sarm1*^*−/−*^ axons from cultured neurons harvested from 3 independent litters were quantified. (**e**) Fluo4-AM calcium imaging of *Wld*^*s*^ sympathetic axons 1 h after injury in the presence and absence of CN03. Scale bar = 10 µm. (**f**) Quantification of axonal spheroid number per 100 µm of *Wld*^*s*^ sympathetic axons at the indicated times after injury in the absence and presence of CN03. Total number of *n* = 25 (Control), *n* = 24 (CN03) *Wld*^*s*^ axons from cultured neurons harvested from 3 independent litters were quantified. (**g**) Representative images and (**h**) quantification of degeneration of wild-type and *Wld*^*s*^ distal sympathetic axons immuno-stained for β3-tubulin with different treatments. Scale bar = 50 µm. For the “NAD^+^” group, axons were incubated in SCG media containing 1 mM NAD^+^ supplement overnight prior to injury. For the “CN03” and CT04″ groups, axons were incubated in SCG media containing 1 µg/mL Rho activator CN03 and Rho inhibitor CT04 for 2 h prior to injury, respectively. Data are reported as mean ± SEM, *p < 0.05; **p < 0.001; ***p < 0.0001, two-way ANOVA with Tukey’s multiple comparisons test.

The Wld^s^ protein stably targets NMNAT1 to axons to maintain high NAD^+^ levels for prolonged periods following injury, which is known to protect axons from degeneration^46^. To test whether NAD^+^ contributes to the formation of axonal spheroids, we treated sympathetic axons with 1 mM exogenous NAD^+^ overnight prior to injury. Supplementation of NAD^+^ to wild-type sympathetic axons suppressed spheroid formation after injury (Fig. 5a,b), and phenocopied observations in *Wld*^*s*^ axons (Fig. 4a,b). We next examined whether NAD^+^ is upstream of Rho activation with respect to regulating spheroid formation. To this end, axons were incubated with the Rho activator, CN03 in the presence of exogenouse NAD^+^. Activation of Rho is able to promote spheroid formation even in the presence of exogenous NAD^+^ (Fig. 5a,b). NAD^+^ levels are known to decline in injured axons and nerves, due to both turnover of NMNAT2 and SARM1-dependent NAD^+^ degradation^18,47,48^. However, loss of *SARM1* failed to inhibit spheroid formation, whereas *Sarm1*^*−/−*^ neurons pre-treated with NAD^+^ or Rho inhibitor CT04 displayed only 1.16 ± 0.52 and 0.85 ± 0.31 spheroids per 100 μm of axons 1 h after injury, respectively (Fig. 5c,d). Moreover, activation of Rho by CN03 in *Wld*^*s*^ neurons increased the number of spheroids to 3.59 ± 0.87 per 100 μm of axons 1 h after injury (Fig. 5e,f). We further examined the protection effects of NAD^+^ in the presence and absence of Rho activation. 4 h after injury, wild-type sympathetic axons treated with exogenous NAD^+^ remained intact, but degeneration was partially rescued (41.7 ± 0.3%) by CN03 incubation (Fig. 5g,h). 8 h after injury, *Wld*^*s*^ axons treated with CN03 displayed 60.5 ± 3.4% degeneration, significantly higher than control *Wld*^*s*^ cultures with 13.8 ± 7.1% degeneration (Fig. 5g,h).

### DR6 and SARM1 act downstream of spheroid formation

**Figure 6:**
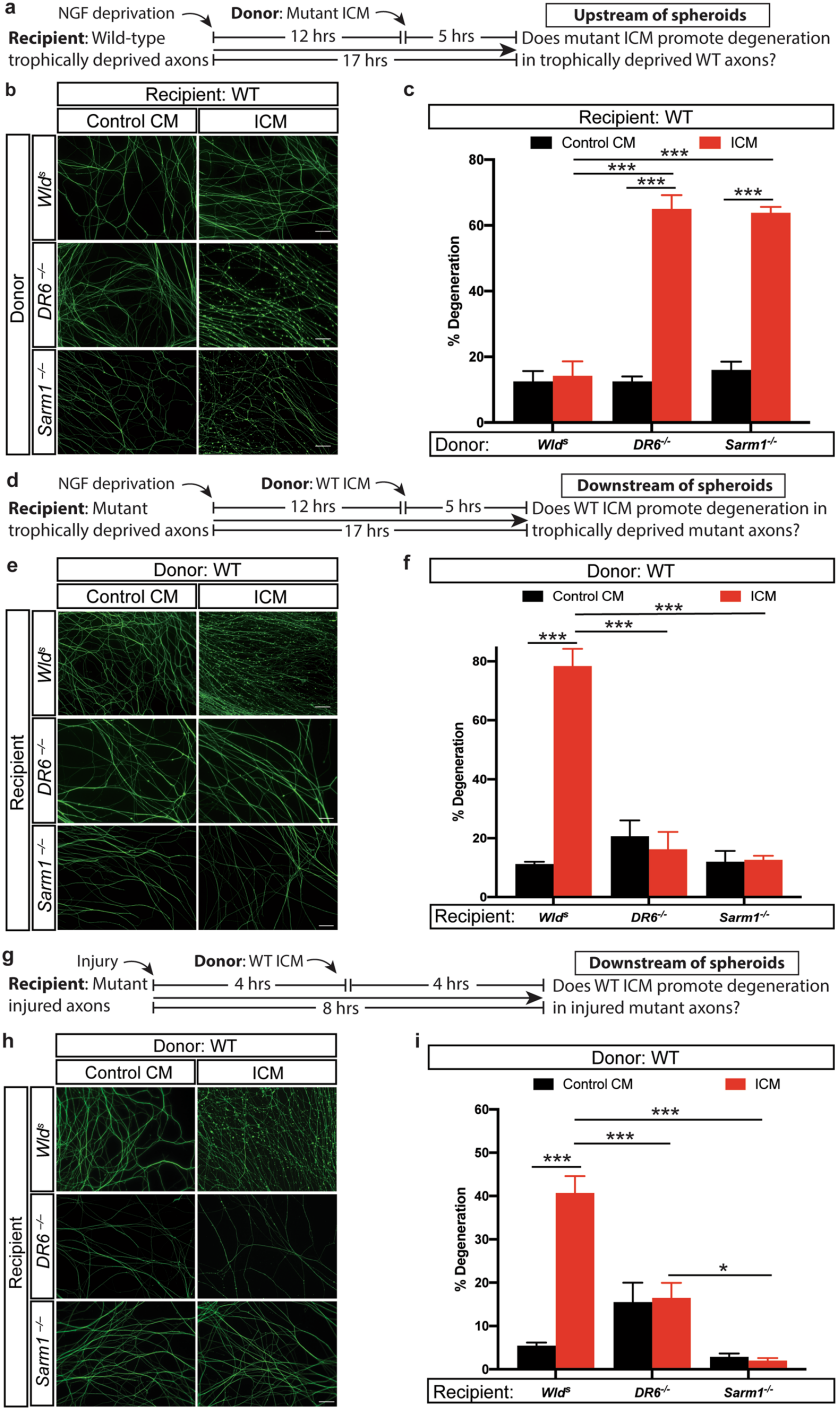
*DR6*^*−/−*^ and *Sarm1*^*−/−*^ suppress ICM induced axon degeneration, while *Wld*^*s*^ does not. (**a**) Wild-type and (**d**) mutant sympathetic neurons were globally deprived of NGF for 12 h followed by addition of conditioned media collected from distal axons for 5 h, respectively. (**b**) Representative images and (**c**) quantification of β3-tubulin immunostained trophic deprived distal sympathetic axons from wild-type animals after treatment with ICM and Control CM collected from *DR6*^*−/−*^*, Sarm1*^*−/−*^ and *Wld*^*s*^ neurons. Compared to *Wld*^*s*^, Control CM (*n* = 4), *p* = 0.9811, *n* = 5 for *Wld*^*s*^, ICM. Compared to *DR6*^*−/−*^, Control CM (*n* = 3), *p* < 0.0001, *n* = 5 for *DR6*^*−/−*^, ICM. Compared to *Sarm1*^*−/−*^, Control CM (*n* = 3), *p* < 0.0001, *n* = 5 for *Sarm1*^*−/−*^, ICM. (**e**) Representative images and (**f**) quantification of β3-tubulin immunostained trophic deprived distal sympathetic axons from *DR6*^*−/−*^*, Sarm1*^*−/−*^ and *Wld*^*s*^ animals after treatment with ICM and Control CM collected from wild-type neurons. Compared to Control CM, *Wld*^*s*^ (*n* = 5), *p* < 0.0001, *n* = 5 for ICM, *Wld*^*s*^. Compared to Control CM, *DR6*^*−/−*^ (*n* = 3), *p* = 0.8921, *n* = 4 for ICM, *DR6*^*−/−*^*.* Compared to Control CM, *Sarm1*^*−/−*^ (*n* = 7), *p* = 0.9988, *n* = 6 for ICM, *Sarm1*^*−/−*^. (**g**) Mutant neurons were injured for 4 h in the presence of NGF followed by addition of conditioned media collected from wild-type axons for 4 h. (**h**) Representative images and (**i**) quantification of β3-tubulin immunostained injured distal sympathetic axons from *DR6*^*−/−*^*, Sarm1*^*−/−*^ and *Wld*^*s*^ animals after treatment with ICM and Control CM collected from wild-type neurons. Compared to Control CM, *Wld*^*s*^ (*n* = 7), *p* < 0.0001, *n* = 11 for ICM, *Wld*^*s*^. Compared to Control CM, *DR6*^*−/−*^ (*n* = 2), *p* = 0.9983, *n* = 4 for ICM, *DR6*^*−/−*^*.* Compared to Control CM, *Sarm1*^*−/−*^ (*n* = 7), *p* = 0.9951, *n* = 8 for ICM, *Sarm1*^*−/−*^. In the “Control CM” group, media was collected from uninjured axons. In the “ICM” group, media was collected 4 h after injury. Data are reported as mean ± SEM, *p < 0.05; ***p < 0.0001. Significant difference is determined by two-way ANOVA with Sidak’s multiple comparison test. Scale bar = 50 µm.

How could injured *Sarm1*^*−/−*^ and *DR6*^*−/−*^ axons remain resistant to degeneration even though they are competent to form and rupture spheroids? We recently showed that loss of *DR6* in trophically deprived axons could prevent catastrophic degeneration downstream of spheroid rupture^10^. Therefore, we hypothesize that SARM1 and DR6 acts downstream of spheroid rupture to promote catastrophic degeneration instead of gating exit from the latent phase of degeneration. To examine this, we collected media surrounding injured distal axons from *Wld*^*s*^*, DR6*^*−/−*^ and *Sarm1*^*−/−*^ cultures and applied this injured conditioned media (ICM) to uninjured trophic deprived wild-type axons (Fig. 6a). These morphologically intact recipient wild-type axons underwent complete degeneration within 5 h of incubation with ICM derived from *Sarm1*^*−/−*^ and *DR6*^*−/−*^ axons (Fig. 6b,c). However, ICM collected from injured *Wld*^*s*^ axons didn’t hasten catastrophic axon degeneration in trophic deprived wild-type neurons (Fig. 6b,c), consistent with the diminished spheroid formation and rupture in these mutants (Fig. 4).

To determine whether SARM1, DR6 or WLD^s^ are downstream of spheroid rupture to regulate degeneration, we applied ICM collected from wild-type axons to intact recipient trophic deprived neurons derived from *Wld*^*s*^*, DR6*^*−/−*^ and *Sarm1*^*−/−*^ mice (Fig. 6d). Remarkably, in this paradigm *Wld*^*s*^ axons showed 78.4 ± 5.8% degeneration, while *DR6*^*−/−*^ and *Sarm1*^*−/−*^ showed 16.3 ± 5.9% and 12.7 ± 1.4% degeneration when exposed to ICM from wild-type axons, respectively (Fig. 6e,f). We next performed a similar experiment on injured recipient axons. We applied wild-type ICM to mutant axons 4 h after injury and then incubate for another 4 h (Fig. 6g). Consistent with the delayed WD in these mutants^25^ , injured *Sarm1*^*−/−*^, *DR6*^*−/−*^ and *Wld*^*s*^ axons displayed less than 20% degeneration 8 h after injury with the final 4 h in the presence of Control CM incubation (Fig. 6h,i). Remarkably, injured *Wld*^*s*^ axons displayed 42.5 ± 9.7% degeneration after WT ICM incubation (Fig. 6h,i). Injured *DR6*^*−/−*^ and *Sarm1*^*−/−*^ axons only showed 16.5 ± 3.5% and 2.0 ± 0.6% when exposed to WT ICM, respectively (Fig. 6h,i). These results raise the possibility that *Sarm1*^*−/−*^ and *DR6*^*−/−*^ protects axons from WD by perturbing pathways required for the catastrophic degenerative response to spheroid contents, while *Wld*^*s*^ delays WD by suppressing the development of axonal spheroids thereby delaying exit from latency.

## Discussion

**Figure 7:**
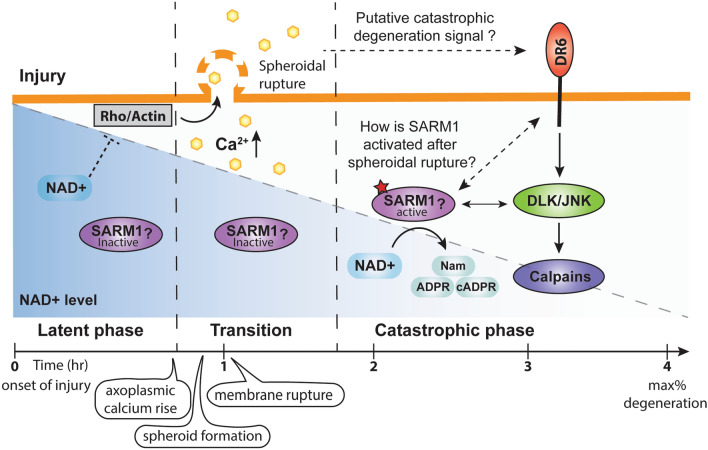
Proposed model for events associated with injury induced axon degeneration of sympathetic neurons. After injury, axoplasmic calcium is increased and enriched in spheroids prior to catastrophic phase. Spheroid formation is regulated by Rho activity and actin remodeling, which is suppressed by NAD^+^. The calcium electrochemical gradient across membrane is disrupted by spheroidal rupture. We speculate that axonal NAD^+^ level decreases via SARM1 independent catalysis while SARM1 stays inactive prior to spheroidal rupture. DR6 and SARM1 can be activated to promote further NAD^+^ depletion and catastrophic degeneration. However, how DR6 and SARM1 get activated downstream of spheroid rupture remains unclear. The schematic representation of the model was drawn in Adobe Illustrator.

Here we describe the regulated formation of calcium rich axonal spheroids as injured axons transition from latent to catastrophic phase of degeneration. Importantly, among three WD deficient mutants, only *Wld*^*s*^ suppresses spheroid formation, suggesting that depletion of axonal NMNAT/NAD^+^ acts upstream of spheroid formation during the latent phase, whereas SARM1 and DR6 activation might promote degeneration during catastrophic phase (Fig. 7). This is somewhat surprising given that the mechanism of SARM1 action is thought to be through NAD^+^ degradation^23,24^. This sets up a scenario whereby NAD^+^ may be acting at different points in the degeneration timeline after injury. It is known that the initial decay of NAD^+^ after injury is independent of SARM1 and we suggest that this reduction is sufficient for the disinhibition of Rho induced spheroid formation. After spheroid rupture, we suggest that DR6 and SARM1 are activated by an as yet unknown mechanism to further drive down the level of NAD^+^ and promote catastrophic axon degeneration (Fig. 7).

Axonal spheroids have been characterized as a common morphological hallmark during axon degeneration^13^. These spheroids arise continuously in axons and show different degrees of swelling in response to a range of molecular triggers, including the focal blockage of axonal transport, ROS mediated actin aggregation, and NMNAT deficiency^38,39,42^. Here, we report the formation of calcium rich spheroids on severed sympathetic axons in vitro (Fig. 1). Similar to our observations in developmental degeneration models^10^, we find that spheroid formation requires Rho dependent actin remodeling. Inhibition of this pathway not only blocks spheroid formation but also delays injury induced degeneration (Fig. 2). However, pharmacological manipulations of actin dynamics or Rho may affect degeneration events other than spheroid regulation, such as remodeling of the actin-spectrin-based membrane associated periodic skeleton, axon transport or ERK signaling^49–52^. Therefore, it’s likely that spheroid formation is the phenotypic result of actin remodeling and Rho activation during axon degeneration, and perhaps an indirect driver of catastrophic fragmentation.

By comparing axoplasmic calcium dynamics of WD deficient mutants with wild-type sympathetic axons, we demonstrated that neurons from *Wld*^*s*^ animals display minimal axonal calcium flux from 20 to 90 min after injury, significantly fewer and smaller axonal spheroids formed, and no calcium extrusion to the extracellular space (Fig. 4). These findings suggest that injury induced NMNAT2 depletion is likely an upstream trigger for calcium rich spheroid formation and rupture. SARM1 has been proposed to be in the same pathway as Wld^s^/NMNAT1 in promoting axon degeneration due to its intrinsic NAD^+^ cleavage activity^23,24,53,54^. Surprisingly, unlike *Wld*^*s*^, *Sarm1*^*−/−*^ axons are capable of forming axonal spheroids after injury suggesting that at least in the context of spheroid formation and rupture, these pathways operate independently. Importantly, this finding is not in conflict with reports that NMNAT/NAD^+^ depletion is involved in catastrophic degeneration^48^. Studies in mouse dorsal root ganglion (DRG) neuron cultures have shown that there is slow NAD^+^ decline loss for roughly 2 h after transection likely owing to NMNAT2 degradation, followed by a fast SARM1-dependent NAD^+^ decay^47,48,55^. To explain the different phenotypes of *Sarm1*^*−/−*^ and *Wld*^*s*^ with respect to spheroid formation after injury, we propose a working model by which SARM1 is inactive during the latent phase and then becomes active after spheroidal rupture to accelerate NAD^+^ depletion during the catastrophic phase of axon degeneration.

The mechanism by which NAD^+^ inhibits Rho activity and spheroid formation remains unknown and will be the subject of future inquiry. We envision a few possibilities: (1) the replenished axonal NAD^+^ pool might inhibit the calcium release from intracellular stores, which contributes to the formation of calcium enriched spheroids. NAD^+^ depletion after injury leads to the increase of relative concentrations of calcium-mobilizing agents including cADPR and ADPR over axonal NAD^+^, stimulating intra-axonal calcium rise by activation of ryanodine receptors on ER and calcium channels on plasma membrane, respectively^56^. In addition, blocking ER calcium channels has been shown to protect injury-induced axonal degeneration in DRG cultures and secondary degeneration of severed CNS axons^57,58^. However, whether blocking intracellular calcium stores would suppress spheroid formation on injured sympathetic axons must be investigated in the future. (2) Depletion of NAD^+^ pools after injury alters axonal redox state and ATP synthesis^59^ which may contribute to spheroid formation and rupture by regulating Rho GTPase activity (Fig. 7). Studies have shown that the cellular oxidation state mediates activation of Rho GTPase via a redox-sensitive cysteine at the end of p-loop motif^60,61^. Moreover, application of Rho activator CN03 is able to promote spheroid formation on injured axons in the presence of NAD^+^ supplementation (Fig. 5a,b). The disruption of redox state or NAD^+^/NADH balance caused by NMNAT2 degradation may therefore activate Rho to mediate actin remodeling and spheroid formation.

The mechanism by which SARM1 is activated after spheroid rupture remains unclear, but the recent finding that NMN analogue can activate SARM1 to induce non-apoptotic cell death appears to provide one possible answer to this question^62^. In a neuroinflammatory model, activation of mixed lineage kinase domain-like pseudokinase (MLKL) can induce loss of axonal survival factors NMNAT2 and SCG10/STMN2 to trigger SARM1 NADase activity, which indicates that necroptotic pathways could disinhibit SARM1 to activate pathological axon degeneration^12,63^. Activation of SARM1 has been shown to promote phosphorylation of JNK to trigger neuronal immune response after axon injury^64^. Moreover, phosphorylation of SARM1 by JNK regulates NAD^+^ cleavage to inhibit mitochondrial respiration in response to oxidative stress^30^. Therefore, SARM1 may also be activated by JNK to promote further NAD^+^ depletion (Fig. 7). Both overexpression of NMNATs and knocking out SARM1 have been shown to decrease injury-induced degradation of the calpain inhibitor, calpastatin, which protects neurons from degeneration^8,65^. It is possible that NAD^+^ depletion and further energy deficits lead to calpastatin degradation, which would disinhibit calpain to promote catastrophic degeneration. Similar to the phenotype of *Sarm1*^*−/−*^, injured *DR6*^*−/−*^ axons showed formation of spheroids and spheroidal calcium accumulation (Fig. 4). Indeed, we’ve shown in the past that DR6 is also required for JNK activity after injury^25^. Because of our previous work examining the role of DR6 in trophic withdrawal induced degeneration, it is tempting to speculate that DR6 gates entry into the catastrophic phase WD by activating SARM1^10^. Injured *Sarm1*^*−/−*^ and *DR6*^*−/−*^ axons are competent for spheroid formation and rupture yet still degenerate much later than injured wild-type axons (Fig. 4). Remarkably, *Sarm1*^*−/−*^ or *DR6*^*−/−*^ neurons, but not *Wld*^*s*^ neurons are resistant to degeneration induced by ICM collected after spheroidal rupture (Fig. 6). Based on these results, we propose that SARM1 and DR6 are likely to promote WD by regulating signaling pathways downstream of spheroid formation and rupture. However, whether and how DR6 and SARM1 would work together to do this is unclear (Fig. 7).

Spheroid formation is a common hallmark for many neurodegenerative disorders including Alzheimer’s disease (AD), glaucoma, amyotrophic lateral sclerosis (ALS)^39,41,66–68^. Our previous work suggests that these spheroids play a functional role as axons transition from latent to catastrophic phases of degeneration^10^. Recently, overactivation of calcium influx in neurons has been shown to trigger degeneration in *Sarm1*^*−/−*^ zebrafish in vivo, suggesting that calcium could be one of the effectors downstream of Sarm1 to drive degeneration^69^. However, we only observed minor attenuation of spheroidal calcium flux in injured *Sarm1*^*−/−*^ and *DR6*^*−/−*^ axons (Fig. 4), indicating that SARM1 and DR6 likely promotes WD independent of calcium signaling. While the pathways downstream of spheroid formation require further investigation, it is intriguing to speculate that the rupture of these spheroids and release of their contents may recruit macrophages and/or modulate Schwann cell injury response. Interestingly, *Sarm1*^*−/−*^ and *Wld*^*s*^ failed to affect macrophage recruitment after injury, while macrophages in injured *Wld*^*s*^ nerve stump showed ‘nerve scanning’ behavior, elongating and extending their process along the distal nerve before fragmentation^70,71^. Lack of spheroids along *Wld*^*s*^ axons might contribute to macrophage scanning behavior as they are looking for the potential targets to engulf. Whether the formation and rupture of axonal spheroids mark the location for phagocytic cells to react or trigger other immune responses during WD remains unclear. As such, understanding the regulation and consequence of these spheroids may help to rationalize therapeutic targets for a range of degenerative disorders.

In summary, this study demonstrates that: (1) severed sympathetic axons develop calcium rich spheroids and membrane ruptures prior to catastrophic degeneration. (2) Mechanistically, we show that sufficient NAD^+^ pool is able to suppress the formation of axonal spheroids and delay WD after injury through a Rho-dependent pathway. 3) DR6 and SARM1 do not regulate spheroid formation, but are required for catastrophic degeneration downstream of spheroidal rupture. Based on our results and recent findings, we propose that 4) NMNAT degradation-dependent NAD^+^ depletion contributes to spheroid formation, while SARM1 activation-dependent NAD^+^ hydrolysis executes axon degeneration after spheroidal rupture in response to injury. Our findings contribute to further understanding of protective NAD^+^ mechanisms in regulating the development of axonal spheroids that aid in the application of WD-blocking therapies for neurodegenerative disorders.

## Methods

### Chemicals and reagents

Fluo-4 (F14200), Fluo-4 AM (F14201), Dextran Texas Red, 3,000, 10,000, and 70,000 MW (D3329, D1828, and D1830, respectively) were purchased from Thermo Fisher Scientific. CT04 and CN03 were the products of Cytoskeleton Inc. Cytochalasin D (C2618), cytosine arabinofuranoside (Ara-C), poly-D-lysine, and paraformaldehyde were purchased from Millipore Sigma. NGF was purified from mouse salivary glands. Goat anti-mouse Alexa 488, 10% goat serum, and laminin (1 μg/mL) were products of Life Technologies. Mouse Tuj1 primary antibody was the product of Covance. DMEM high glucose (11,965,092), DMEM/F-12 (11,320,033), DMEM/F-12 phenol red free (21,041,025), DMEM high glucose, calcium free (21,068,028), 100X penicillin/streptomycin (10,378,016), and FBS (16,000,044) were brought from Gibco.

### Animals

All experiments were carried out in compliance with the Association for Assessment of Laboratory Animal Care policies and approved by the University of Virginia Animal Care and Use Committee. All mice were on a C57BL/6J.129S mixed background except for *Wld*^*s*^ mice, which were FVB/NJ background. *Wld*^*s*^ and *Sarm1*^*−/−*^ animals were purchased from the Jackson labs. *DR6*^*−/−*^ animals were a generous gift from Genentech. Males and females were mixed in all experiments.

### Primary sympathetic neuronal cultures

Sympathetic neuron cultures were established as described previously^72^. Briefly, neurons were obtained by dissociation of P0-P2 mouse superior cervical ganglia. These neurons (from each litter of pups) were plated in compartmentalized microfluidic devices coated with poly-D-lysine (50 μg/mL) and laminin (1 μg/mL) in DMEM supplemented with 10% FBS, penicillin/streptomycin (1 U/mL), and 45 ng/mL of NGF at 37 °C. Glial cells were removed from cultures using 5 μM Ara-C for 48–72 h.

### Axotomy experiments in vitro

Neurons from each litter of pups were allowed to project their axons to the axonal chamber (3–7 DIV) after plating. After the axons had grown into the axonal chamber, neurons were enucleated by aspirating 3 mL of 1 × PBS through the cell body chamber leaving the axons intact in their respective chamber. Unless otherwise indicated, both compartments were replaced with DMEM supplemented with 10% FBS, penicillin/streptomycin (1 U/mL) and 45 ng/mL of NGF and incubated at 37 °C and 10% CO_2_ for indicated times. For technical consistency, we used encleation in microfluidic chambers for injury. In our experimental system, axons originating from wild-type dissociated SCG neurons degenerate within 2 h after injury, while neurites originating from SCG explant degenerate within 8 h after scalpel cut^73^. The difference in axon degeneration time window could be due to dissociation procedure and time in culture. We suspect that the most likely explanation for this difference is in the media volume of these cultures, where spheroid derived pro-degenerative molecules are more concentrated in the 200 μL volume of an MFD as opposed to the 1–3 mL volume bathing explants. Nevertheless, the morphological changes from latent to catastrophic phase, as well as the calcium wave in “physical cut method” remain the same as what we observed in our model^73^.

### Conditioned media experiments

Established compartmentalized recipient neuron cultures were maintained in the presence of NGF. For global NGF deprivation, cultures were washed with NGF-free DMEM three times and incubating at 37 °C with neutralizing anti-NGF antibody for 12 h. To collect injury conditioned media (ICM), donor neuron cultures were injured by PBS aspiration in cell body chamber. For each device, a volume of ~ 100 μL of ICM was collected, including the media in the wells of the device on the axonal side and the media in the axonal channel. ICM or control conditioned media were applied on recipient axons for 4 or 5 h.

### Immunocytochemistry

Immunocytochemistry was carried out as previously described^74^. Briefly, at the indicated times, axons were fixed in 4% paraformaldehyde (w/v)/ phosphate buffered saline (PBS) at room temperature for 20 min, washed 3 × 5 min with 1 × PBS, and blocked/permeabilized (5% goat serum, 0.05% Triton-x-100 in PBS) for 1 h at room temperature. Axons were then incubated overnight at 4 °C with primary antibody diluted in the blocking buffer. Cells were then washed 3 × 5 min with 1 × PBS and incubated with fluorescent secondary antibody for 1 h at room temperature. Cells were again washed with 1 × PBS three times and imaged using a fluorescent inverted microscope. All in vitro experiments were performed in triplicate with at least two microfluidic devices used for each condition.

### Live imaging

Sympathetic neuron cultures were washed 3 times with DMEM/F-12, phenol red free, and incubated for 30 min at 37 °C and 10% CO_2_ with live imaging dyes diluted in DMEM/F-12, phenol red free. Cells were then imaged under Leica SP5 X confocal microscope in W.M. Keck Center at the University of Virginia. Axons in grooves of the microfluidic chamber were imaged after injury. For membrane rupture, dextran dyes diluted in DMEM/F-12, phenol red free were added to the microfluidic chamber 20 min after injury.

### Image processing and analysis

Axon degeneration in culture was quantified from β3-tubulin stained fluorescence images by counting the number of individual axons at the leading edge that had at least three beads/blebs as described^26^. A blinded investigator counted ten representative pictures of the axons, in two microfluidic chambers per condition/time point. On each image 10 50 μm boxes were randomly assigned to single axons. The investigator took care not to box bundles of axons, which may confound analysis. Then the number of boxes, which had 3 or more beads/blebs were counted and categorized as degenerating axons. Equal to or more than 80% degeneration was considered maximum degeneration and equal to or less than 10% degeneration of axons was considered as minimum degeneration. The percentage of the total number of degenerating axons was calculated using Microsoft Excel. At least 300 total axons were counted for each condition. The standard error of the mean was considered as error. In live imaging, Ca^2+^ intensity (ΔF/F0), the size (S/S0) and number of axonal spheroids were quantified in selected ROI (single axon or axonal spheroid) by Fiji software^75^. Each experiment was repeated at least 3 times with separate litters of mouse pups of the same genotype.

### Calcium measurement

Conditioned media was diluted in milliQ water (1:20) and mixed thoroughly. 100 μL of reaction mixture was made with 1 μL HEPES, 2 μL 1 mM Fluo-4 (20 mM working concentration), 10 μL diluted conditioned media, and 87 μL water. Black 96 well plate was used in Spectrophotometric assay. Eight CaCl_2_ standards (2.54 μM, 4.87 μM, 9.75 μM, 19.5 μM, 39 μM, 78 μM, 156 μM, and 313 μM) were used to calculate a standard curve for analyzing Ca^2+^ concentration in conditioned media.

### Statistics

Statistical analysis was performed in Prism 8.0 software (GraphPad). All measurements are shown as mean ± SEM. For samples defined by one factor, data were compared by unpaired two-tailed *t* tests for two samples or one-way ANOVA with Tukey’s post hoc multiple comparisons test for three or more samples. For samples defined by two factors, data were compared by two-way ANOVA with Sidak’s or Dunnett’s post hoc multiple comparisons test. Sample size (n) was defined as the number of axons or axonal spheroids counted in the live imaging experiment, or the number of independent cultures that were quantified in each experiment. The null hypothesis was rejected at the 0.05 level. *p* values < 0.05 were considered significant and represented by asterisks. The statistical test, sample size (n), and the *p* values were reported in the figure legends.

### Ethical approval

All procedures for animal use were carried out in compliance with Association for Assessment of Laboratory Animal Care policies and approved by the University of Virginia Animal Care and Use Committee.

### Consent for publication

We approve the manuscript for publication after acceptance.

## Supplementary information

Supplementary Information.

Supplementary Legends.

## Data Availability

The raw datasets used and/or analyzed during current study are available from the corresponding author on reasonable request.
