# Supplementary material for: Regulation of degenerative spheroids after injury
Source: Sci Rep. 2020 Sep 22;10:15472. doi: 10.1038/s41598-020-71906-x (PMC7508847; doi:10.1038/s41598-020-71906-x)
Supplement: Supplementary file 2 — Supplementary Legends. [file 41598_2020_71906_MOESM2_ESM.docx]

**Regulation of degenerative spheroids after injury**

Yu Yong^1^, Kanchana Gamage^2^, Courtny Cushman^3^, Anthony Spano^1^, Christopher Deppmann^1,3*^

^1^Department of Biology, University of Virginia, Charlottesville, VA, 22903, USA

^2^Amgen, Massachusetts & Department of Stem Cell and Regenerative Biology, Harvard University, Cambridge, MA, 02138, USA

^3^Department of Neuroscience, University of Virginia, Charlottesville, VA, 22903, USA

***To whom correspondence should be sent:**

Christopher D. Deppmann

Departments of Biology, Cell Biology, Neuroscience and Biomedical Engineering

University of Virginia

Charlottesville, VA 22904-4328

email: [deppmann@virginia.edu](mailto:deppmann@virginia.edu)

**Supplementary video 1: Dextran 3 kDa entry to axonal spheroids after injury**

Live imaging of dextran 3 kDa (red) entry to axonal spheroids (black) 40 minutes after injury. Yellow empty arrows indicate that dextran 3 kDa entry to axonal spheroids at later time points. White filled arrows indicate spheroid moving and merging to form a bigger spheroid that eventually ruptured. No vesicular or punctate labeling was observed during dextran incubation, which is consistent with membrane rupture, instead of macropinocytosis. Scale bar = 10µm.
